# Differential requirement of F-actin and microtubule cytoskeleton in cue-induced local protein synthesis in axonal growth cones

**DOI:** 10.1186/s13064-015-0031-0

**Published:** 2015-02-25

**Authors:** Michael Piper, Aih Cheun Lee, Francisca PG van Horck, Heather McNeilly, Trina Bo Lu, William A Harris, Christine E Holt

**Affiliations:** Department of Physiology, Development and Neuroscience, University of Cambridge, Downing street, Cambridge, CB2 3DY UK; Current address: The School of Biomedical Sciences and the Queensland Brain Institute, The University of Queensland, St Lucia, QLD 4072 Australia; Current address: Institute of Neuroscience, Chinese Academy of Sciences, Shanghai, 200031 China

## Abstract

**Background:**

Local protein synthesis (LPS) via receptor-mediated signaling plays a role in the directional responses of axons to extrinsic cues. An intact cytoskeleton is critical to enact these responses, but it is not known whether the two major cytoskeletal elements, F-actin and microtubules, have any roles in regulating axonal protein synthesis.

**Results:**

Here, we show that pharmacological disruption of either microtubules or actin filaments in growth cones blocks netrin-1-induced *de novo* synthesis of proteins, as measured by metabolic incorporation of labeled amino acids, implicating both elements in axonal synthesis. However, comparative analysis of the activated translation initiation regulator, eIF4E-BP1, revealed a striking difference in the point of action of the two elements: actin disruption completely inhibited netrin-1-induced eIF4E-BP1 phosphorylation while microtubule disruption had no effect. An intact F-actin, but not microtubule, cytoskeleton was also required for netrin-1-induced activation of the PI3K/Akt/mTOR pathway, upstream of translation initiation. Downstream of translation initiation, microtubules were required for netrin-1-induced activation of eukaryotic elongation factor 2 kinase (eEF2K) and eEF2.

**Conclusions:**

Taken together, our results show that while actin and microtubules are both crucial for cue-induced axonal protein synthesis, they serve distinct roles with F-actin being required for the initiation of translation and microtubules acting later at the elongation step.

**Electronic supplementary material:**

The online version of this article (doi:10.1186/s13064-015-0031-0) contains supplementary material, which is available to authorized users.

## Background

The ability of neuronal growth cones to synthesize proteins locally helps to control various aspects of axon growth and guidance. Turning and collapse responses [1-9], chemotropic adaptation [3,10,11], and the local upregulation of specific receptors and adhesion molecules allowing growth cones to move on from intermediate targets [12-15] all involve local protein synthesis (LPS). LPS depends on targeting mRNAs to the correct subcellular location and on the local activation of translational machinery, thus ensuring the synthesis of particular proteins at the proper time and place. In developing retinal ganglion cells (RGCs) in *Xenopus*, the guidance cue netrin-1 triggers the transport of the zipcode binding protein 1 (ZBP1; known as Vg1RBP in *Xenopus*) into growth cone filopodia [5]. ZBP1 is responsible for the localization of several species of mRNA, in part, by binding to their *cis*-acting ‘zipcodes’ which are located in the 3′ untranslated regions (3′ UTRs) of mRNA targets, and together, they are transported as translationally dormant ribonucleoparticles (RNPs) [16]. ZBP1 and one of its target mRNAs, β-actin, become asymmetrically localized to the proximal side of growth cones when exposed to a polarized netrin-1 gradient [5,7], and this cue-proximal side shows elevated levels of β-actin protein synthesis within minutes [5]. Inhibition of β-actin translation blocks attractive growth cone turning without affecting extension indicating a key role for LPS in cue-induced directional steering [5,7].

The movement of RNPs and the graded process of translational activation suggest a role for components of the cellular cytoskeleton in LPS. It is known that long-range mRNA distribution into axons and dendrites occurs through motor-based transport on microtubule tracks [17,18], and dispersion into dendrites and dendritic spines is thought to be mediated by actin-based myosin transport [18-20]. Early electron microscope studies in various non-neuronal cell types show polysomes attached to cytoskeletal filaments [21-24]. mRNAs and several factors of the translation machinery have also been found to associate with the cytoskeleton, and the degree of this association can be modulated by physiologically relevant stimuli [25,26]. Thus, it seems plausible that components of the cytoskeleton may play critical roles in the rapid, highly localized translational responses of growth cones to cue-induced stimuli. Surprising, however, this has not yet been tested.

We sought, therefore, to investigate the roles of the F-actin and microtubule cytoskeleton in guidance cue-induced translation in the growth cone by disrupting either F-actin or microtubules while examining netrin-1-induced LPS. Interestingly, both cytoskeletal elements are required for LPS, but each one regulates distinct aspects of this process: actin microfilaments are needed for the early signaling steps in a growth cone’s response to netrin-1 stimulation leading to translation initiation, whereas microtubules are not required until after the translation initiation step.

## Results

### F-actin and microtubule inhibitors block netrin-1-induced local protein synthesis

**Figure 1:**
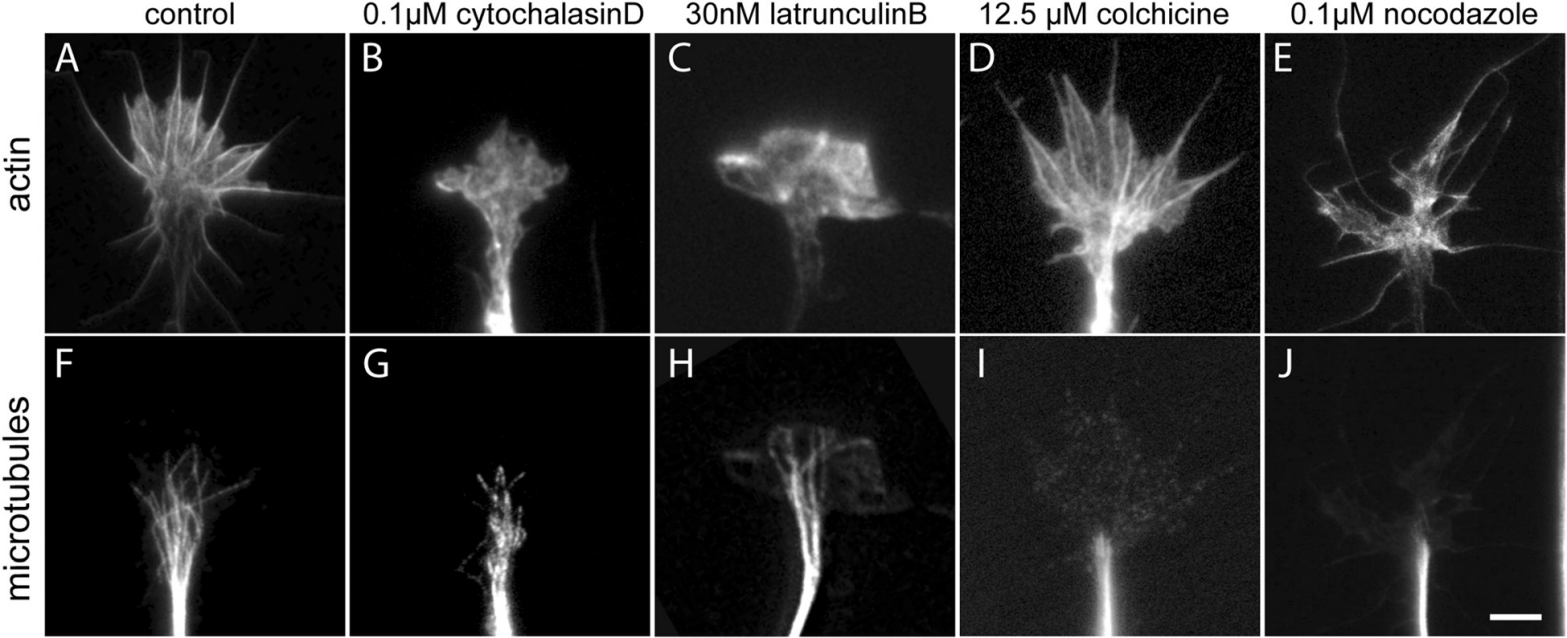
**The effects of pharmacological inhibitors of actin and microtubule polymerization on retinal growth cone morphology.** Embryonic eyes at stage 24/25 were cultured for 24 h, treated with either control medium or pharmacological inhibitors for 5 min, and stained for F-actin (Phalloidin-Alexa488) or β-tubulin. Control retinal growth cones **(A)** contained an extensive network of actin filaments, which was disrupted upon treatment with **(B)** cytochalasin D (0.1 μM) and **(C)** latrunculin B (30 nM). Similarly, control retinal growth axons show microtubules in the growth cone central domain and individual microtubules invading the peripheral domain **(F)**. The dynamic microtubules in the growth cone, but not in the axon shaft, were depolymerized upon treatment with **(I)** colchicine (12.5 μM) or **(J)** nocodazole (0.1 μM). Importantly, this treatment paradigm of actin or microtubule inhibitors did not affect the ultrastructure of the other cytoskeletal system** (G and** **H,** **D and** **E)** and also did not increase growth cone collapse at the concentrations used. Scale bar 10 μm.

To investigate the role of the growth cone cytoskeleton in guidance cue-induced translation, F-actin or microtubule dynamics were disrupted in the growth cone using the pharmacological inhibitors cytochalasin D and latrunculin B (inhibitors of actin polymerization) and colchicine and nocodazole (inhibitors of microtubule polymerization). Since high doses of these drugs can disrupt both cytoskeletal elements, we first conducted dose-response experiments to determine the lowest dose of inhibitor that disrupted one cytoskeletal element without affecting the other. Drug-treated growth cones were dual-stained using Alexa-fluor-phalloidin (Invitrogen Life Technologies, Carlsbad, CA, USA) (F-actin) and anti-β-tubulin and were assessed with fluorescence microscopy coupled with digital image capture. Our results showed that 0.1 μM of cytochalasin (or 30 nM latrunculin B) disrupted the actin cytoskeleton without affecting the microtubules in the growth cone (Figure 1A, B, C, F, G, H). Similarly, we found that 12.5 μM of colchicine (or 0.1 μM nocodazole) left the actin cytoskeleton intact while completely disrupting growth cone microtubules (Figure 1D, E, I, J).

**Figure 2:**
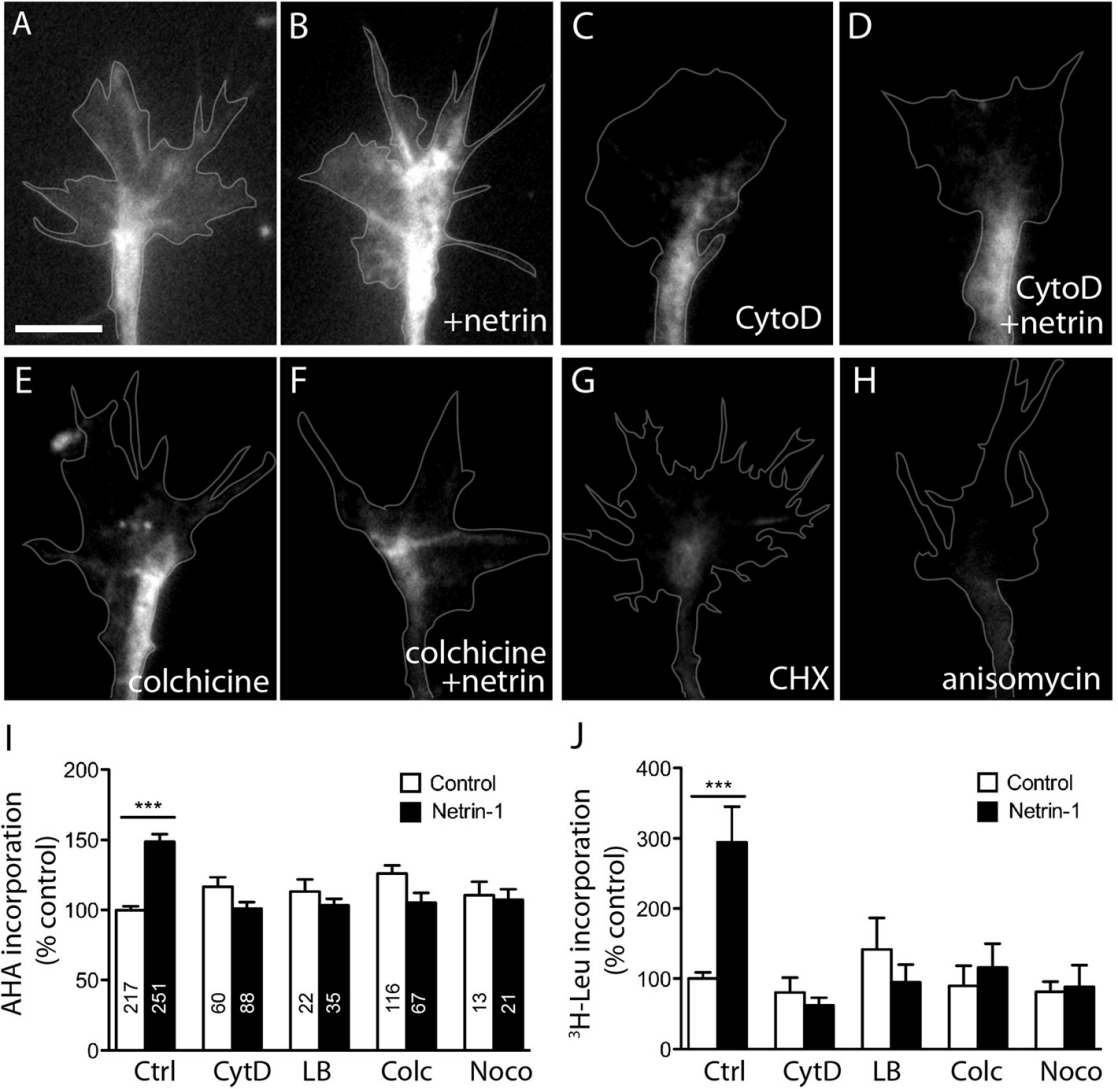
**Intact actin and microtubule cytoskeleton are both required for netrin-1-induced protein synthesis.** Stage 24/25 retinal explants were treated with control medium, cytochalasin D (CytoD), latrunculin B (LB), colchicine (Colc), or nocodazole (Noco) for 5 min, followed by stimulation with either control medium or netrin-1 for a further 5 min. Protein synthesis in growth cones was measured by methionine analog L-azidohomoalanine (AHA) incorporation and visualized with fluorescent microscopy **(A-I)**. Protein synthesis in growth cones treated with control medium **(A)** was markedly increased 5 min following treatment with netrin-1 **(B)**. However, this increase was abolished in growth cones treated with cytochalasin D **(C, D)** or colchicine **(E, F)**. Protein synthesis within the growth cone was almost completely abolished by pretreatment with cycloheximide (CHX, G) or anisomycin **(H)**. Quantification of fluorescence intensity reveals that netrin-1-induced protein synthesis is inhibited by disruption of either actin or microtubule dynamics **(I)**. The number of growth cones analyzed in each treatment group can be found in the corresponding bar of the graph in panel I. Similarly, protein synthesis, as measured by the incorporation of ^3^H-leucine in precipitated proteins, was stimulated by netrin-1, but this effect was inhibited by treatment with either cytoclalasin D, latrunculin B, colchicine, and nocodazole **(J)**. ***P < 0.0001 Mann-Whitney test. Scale bar 10 μm.

We next asked whether these cytoskeletal inhibitors can affect LPS. To measure LPS, we used a metabolic labeling method, the incorporation of L-azidohomoalanine (AHA). AHA is a methionine analog that can be covalently coupled to an alkyne-conjugated fluorochrome via click chemistry [27,28], and we used this fluorescent non-canonical amino acid tagging (FUNCAT) system as a reporter method to visualize newly synthesized proteins within growth cones [29]. In cultured stage 24/25 *Xenopus* retinal ganglion cell neurons, we found that FUNCAT is a robust method to detect new protein synthesis, as cells treated with the protein synthesis inhibitors cycloheximide and anisomycin displayed near background levels of FUNCAT signal in comparison to untreated controls (Figure 2A, G, H). We then performed FUNCAT on growth cones treated with cytoskeletal inhibitors and stimulated the cultures with 600 ng/μl netrin-1 for 5 min. Netrin-1 bath application significantly increased the FUNCAT signal in growth cones, and this increase was completely blocked by either actin or microtubule disruption (Figure 2A, B, C, D, E, F, I).

To validate these results, we performed a second metabolic labeling assay, namely the incorporation of ^3^H-leucine, on surgically isolated retinal growth cones [1]. Growth cones severed from their cell bodies were treated with the cytoskeletal inhibitors followed by concurrent addition of ^3^H-leucine and netrin-1. Tri-chloroacetic acid was used to precipitate proteins *in situ* on the coverslips, and isotope uptake was measured with a scintillation counter. Ten minutes of netrin-1 stimulation led to a significant increase in ^3^H-leucine uptake compared to control conditions (Figure 2J and [1], whereas cells treated with either actin (cytochalasin D or latrunculin B) or microtubule disrupting drugs (colchicine or nocodazole) did not show a significant change in isotope uptake when compared to controls (Figure 2J). Thus, the integrity of both actin and microtubule cytoskeletal systems is required for the local synthesis of proteins in growth cones.

### F-actin, not microtubules, required for early steps in netrin-1-induced LPS pathway

**Figure 3:**
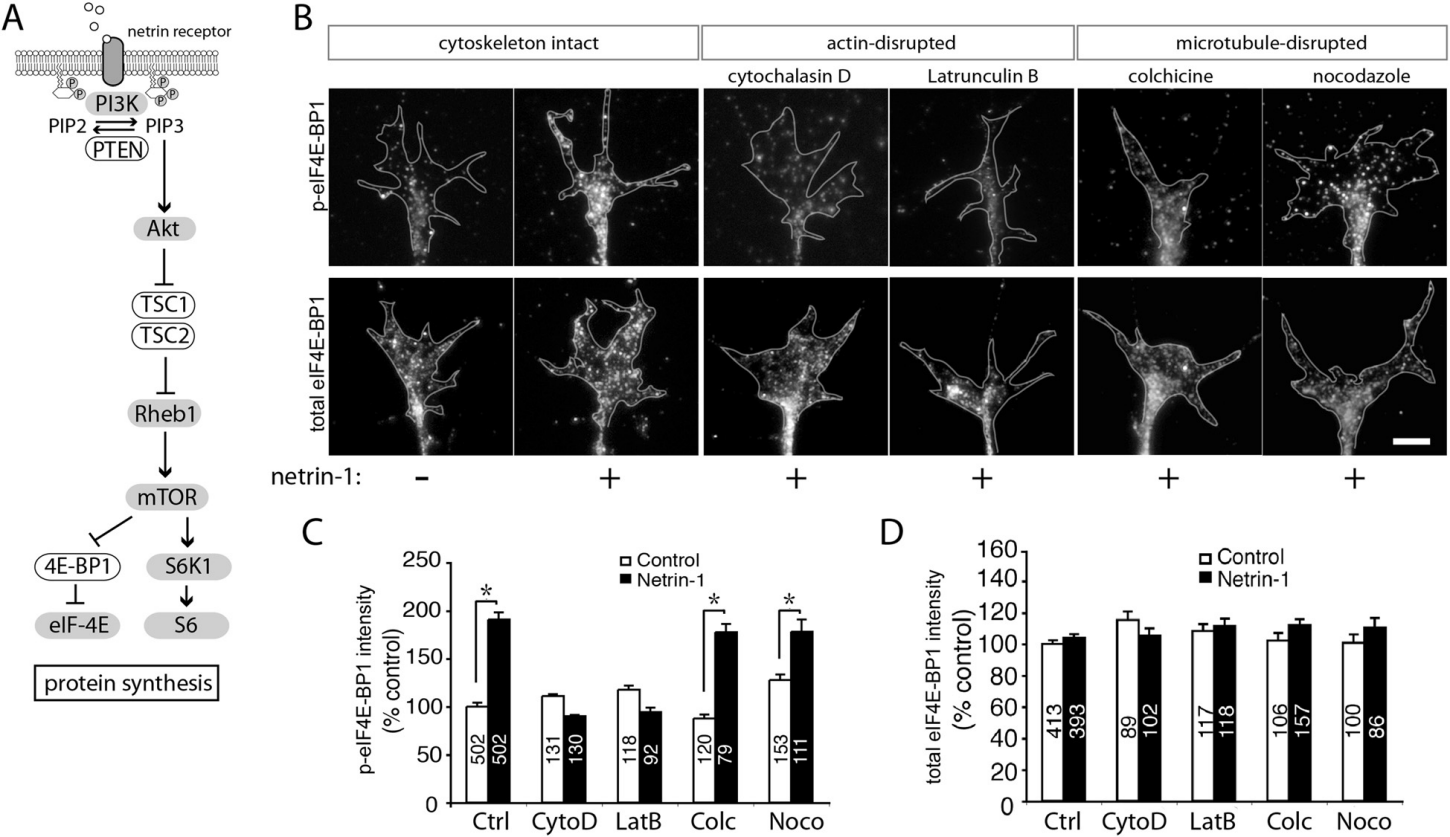
**Netrin-1-induced increase in eIF4E-BP1 phosphorylation requires an intact actin but not microtubule cytoskeleton.** A brief schematic of signaling from netrin receptor leading to protein synthesis **(A)**. Stage 24/25 retinal explants were cultured for 24 h, then treated with the actin depolymerizing agents cytochalasin D (CytoD) and latrunculin **(B)** (LatB), or the microtubule depolymerizing agents colchicine (Colc) and nocodazole (Noco) for 5 min, followed by stimulation with either control medium or netrin-1 for a further 5 min. To assess the activation of translation, retinal cultures were stained for phospho-eIF4E-BP1 (p-eIF4E-BP1; growth cones in the top row of panel **(B)**) and the intensity of the immunofluorescence signal was measured per unit area (quantified in **(C)**). Compared to the control (top row of** (B)**, far left panel) netrin-1 induced a significant increase in P-eIF4E-BP1 signal intensity (top row of (B), cytoskeleton intact), which was completely blocked upon pretreatment with actin inhibitors (top row of **(B)**, actin-disrupted), but not with microtubule inhibitors (top row of **(B)**, microtubule-disrupted. Quantification of p-eIF4E-BP1 signal intensity is shown in **(C)**. *P < 0.05 Mann-Whitney test. In contrast, levels of total eIF4E-BP1 signal intensity (bottom row of **(B)**) were not significantly affected by netrin-1 or the pharmacological inhibitors. The number of growth cones analyzed in each treatment group can be found in the corresponding bar of the graphs in panels** (C)** and **(D)**. Scale bar 10 μm.

Protein synthesis is a multistep process that involves the assembly of dozens of translation machinery components [30,31]. Therefore, we next asked which steps of this process require intact actin and microtubules. The phosphorylation of the translation initiation factor regulator eIF4E-binding protein 1 (eIF4E-BP1) is a key event in cap-dependent translation initiation (Figure 3A) [32], and phospho-eIF4E-BP1 immunostaining can be used as a reporter for translation initiation. Stimulation of retinal growth cones with the guidance cue netrin-1 has previously been shown to trigger rapid phosphorylation of eIF4E-BP1, culminating in LPS [1]. Quantitative immunofluorescence (QIF) of growth cones from stage 24/25 retinal explant cultures showed that phospho-eIF4E-BP1 levels increased nearly twofold 5 min following stimulation with netrin-1 (600 ng/ml). When retinal growth cones were pretreated with cytoskeletal drugs for 5 min, the increase in phospho-eIF4E-BP1 levels was preserved in colchicine or nocodazole-treated growth cones but, significantly, it was abolished in growth cones treated with cytochalasin D and latrunculin B (Figure 3B, C). These results cannot be explained by a change in the volume of the growth cone as total eIF4E-BP levels remained similar under netrin-1-stimulated and drug-treated conditions (Figure 3D). Thus, translation initiation in response to netrin-1 stimulation requires an intact F-actin cytoskeleton, but not intact microtubules, in the neuronal growth cone.

**Figure 4:**
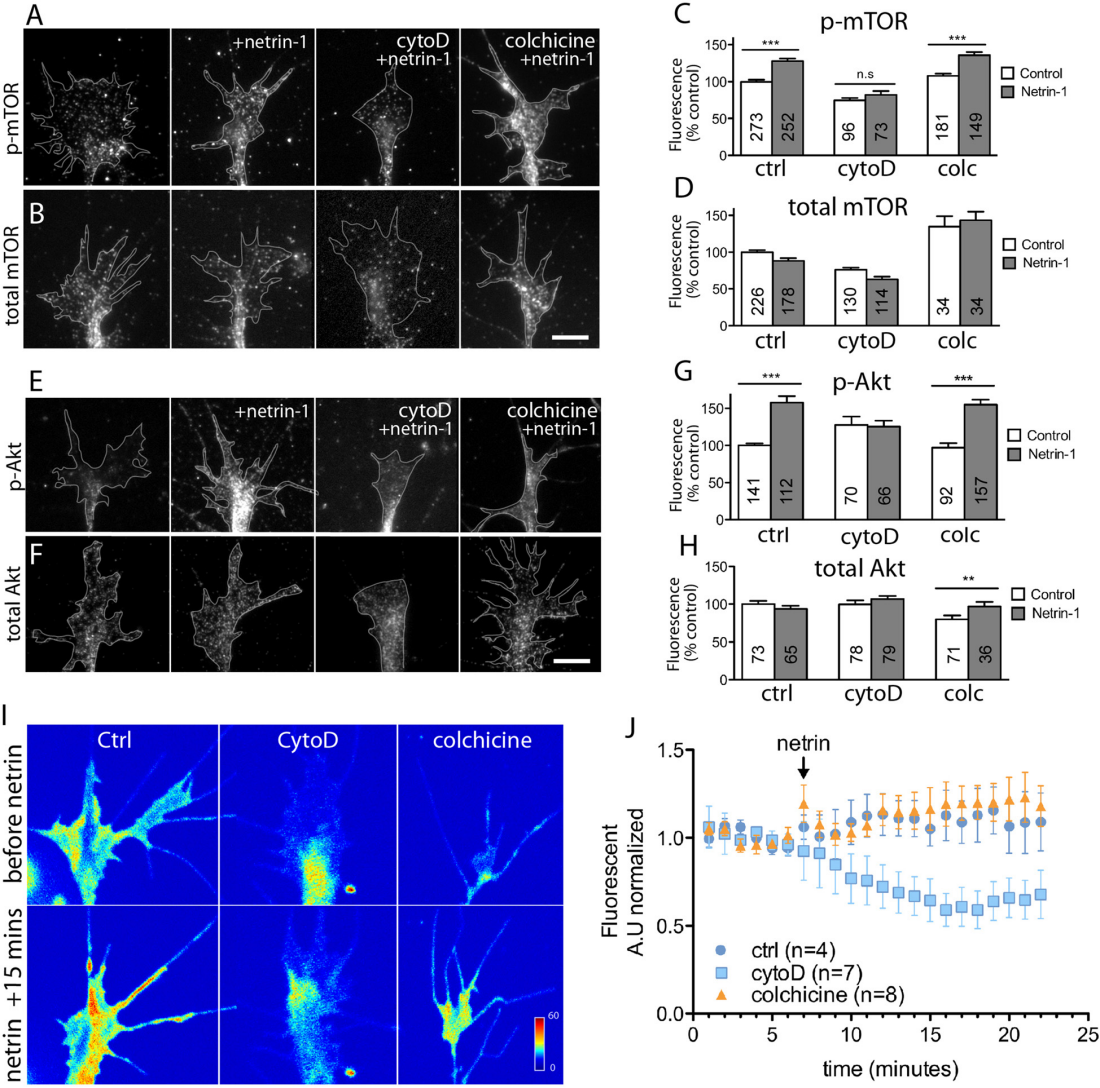
**Actin, but not microtubule, disruption blocks netrin-1 induced PI3K/Akt/mTOR signaling in growth cones.** Cultured stage 24/25 retinal explants were treated with a DMSO vehicle control, cytochalasin D (CytD), or colchicine (Colc) for 5 min, followed by 5-min stimulation with either control medium or netrin-1. Quantitative immunofluorescence showed that levels of activated mTOR (p-mTOR; **(A)**) and Akt (p-Akt; **(E)**) were elevated by netrin-1 stimulation, a process that was prevented by actin, but not microtubule disruption (quantified in **(C)** and **(G)**). Total levels of mTOR **(B)** and Akt **(F)** were not significantly altered following stimulation with netrin-1, in either untreated or growth cones treated with cytoskeletal disrupting agents (quantified in **(D)** and **(H)**), with the exception of total Akt levels in growth cones treated with colchicine, which showed a small, but significant increase in fluorescence intensity. The number of growth cones analyzed in each treatment group can be found in the corresponding bar of the respective graphs. **P < 0.005; ***P < 0.0001 Mann-Whitney test. **(I)** Pseudocolored images of a live PI3K biosensor (PHAkt-GFP) before and after netrin-1 stimulation in the presence of cytoskeletal inhibitors. Growth cones expressing low levels of PHAkt-GFP were treated with cytoskeletal inhibitors (0.1 μM cytochalasin D or 12.5 μM colchicine) on stage during time-lapse acquisition on an inverted spinning disk confocal system (60× water immersion). Time-lapse imaging showed an increase in PHAkt-GFP signal after netrin-1 stimulation, which was inhibited by cytochalasin D treatment, but not affected by colchicine treatment **(J)**. Background-subtracted fluorescent signals were normalized to the control medium at time 0. Scale bar 10 μm.

The phosphorylation of eIF4E-BP1 depends on target of rapamycin (mTOR) activation, which itself is downstream of the phosphoinositide 3-kinase (PI3K)/Akt signaling pathway [33], which is, in turn, activated in response to netrin-1 [1,34-36] (Figure 3A). To investigate whether actin is required for netrin-1-induced signaling upstream of eIF4E-BP1 phosphorylation, we performed immunocytochemistry in neuronal growth cones to detect active and total forms of specific signaling components in the PI3K/Akt pathway. QIF showed that upon netrin-1 stimulation, the phosphorylation (activation) of mTOR and Akt both required intact actin in the growth cones, but, as expected, not microtubules (Figure 4A, B, C, D, E, F, G, H).

Next, we investigated whether PI3K activation also requires the cytoskeleton, and we expressed a live PI3K activity reporter, PHAkt-GFP, in retinal ganglion cell neurons and performed time-lapse imaging of growth cones in response to bath-applied netrin-1 in the presence of cytoskeletal drugs. The biosensor PHAkt-GFP consists of GFP fused to the PIP3-binding PH-domain of Akt [37]. It binds PIP3 in the plasma membrane where it is activated by PI3K [36,38,39]. We expressed PHAkt-GFP in cultured stage 24/25 *Xenopus* eye primordia and selected growth cones with low levels of PHAkt-GFP expression, so that the PH domain did not interfere with endogenous PI3K signaling [40]. Growth cones were imaged at 5-s intervals throughout the following treatment: 2 min of control imaging medium (with 0.1% BSA), 5 min of cytoskeletal drug (DMSO control, 0.1 μM cytochalasin D or 12.5 μM colchicine), 15 min of netrin-1 bath (600 ng/ml, in the presence of cytoskeletal drug). Background-subtracted fluorescence intensity measurements taken from tracings of the thin growth cone peripheral domain (filopodial and lamellipodial regions; [41]) were normalized to *t* = 0 in control-treated growth cones. We were able to detect a mild but steady increase in PHAkt-GFP signal in control growth cones stimulated with netrin-1, which also occurred in microtubule-disrupted growth cones. However, throughout the duration of cytochalasin D treatment, growth cones showed a decline of PHAkt-GFP fluorescence intensity, despite netrin-1 stimulation (Figure 4I, J), indicative of actin cytoskeletal integrity being required for PI3K activation in response to netrin-1 stimulation.

Thus, we have shown that the actin cytoskeleton is required for the activation of the early steps in the guidance receptor-mediated translation cascade, starting with PI3K/Akt and then mTOR and eIF4E-BP1. The microtubule cytoskeleton is not necessary for these early steps but is required for LPS, raising the question of where it acts in the translation pathway.

### Microtubules are involved in polypeptide chain elongation or translation arrest

**Figure 5:**
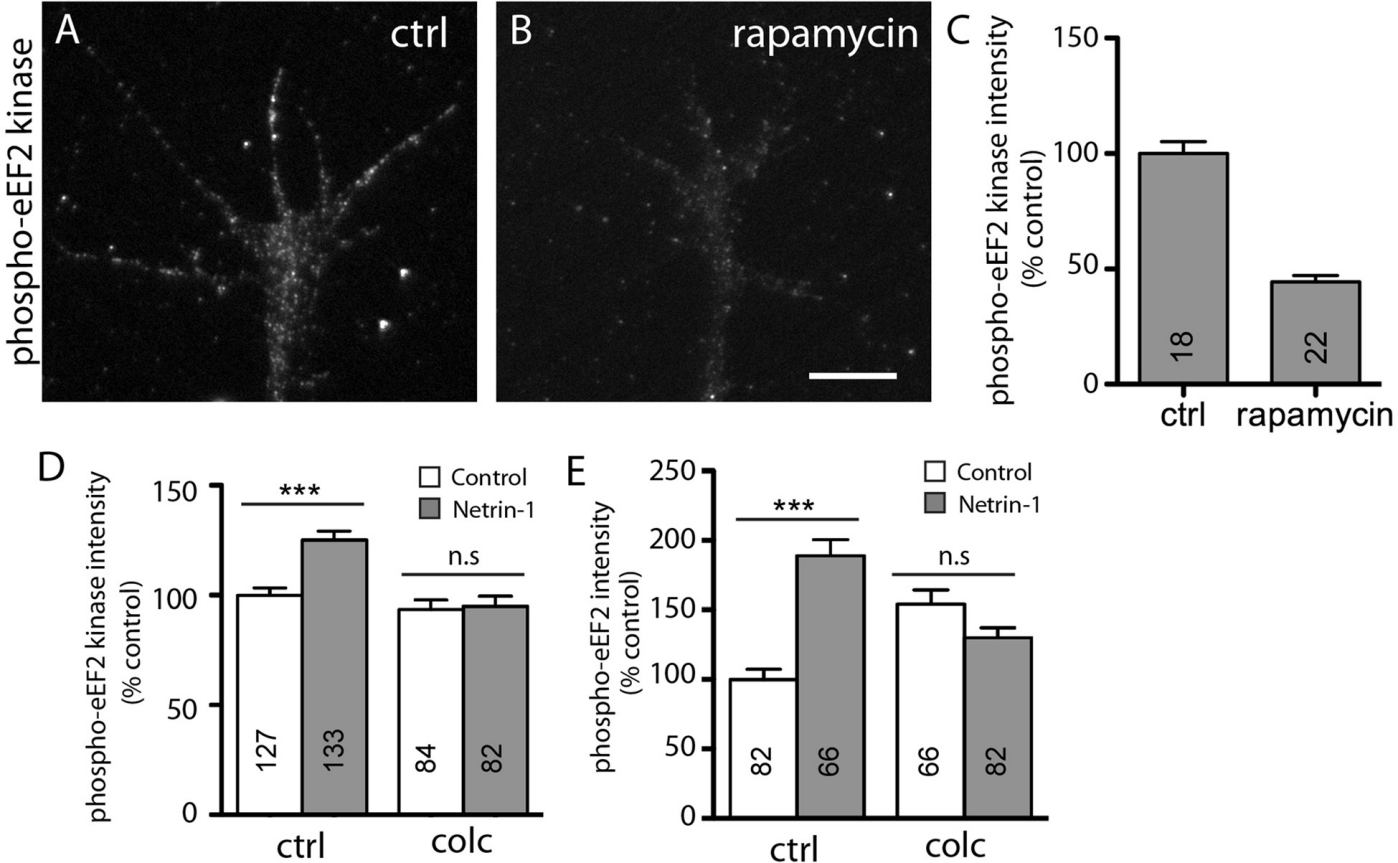
**Microtubule depolymerization inhibits netrin-1 induced phosphorylation of EF2K and EF2.** Cultured stage 24/25 retinal explants were treated with the mTOR inhibitor rapamycin or a vehicle only control **(A, B)**. Rapamycin induced a significant reduction in the level of phosphorylation of a downstream target of mTOR, eEF2 kinase **(A-C)**. Quantitative immunofluorescence was also used to assess the levels of phosphorylation of both eEF2 kinase **(D)** and eEF2 **(E)** in response to netrin-1 stimulation. Netrin-1 induced a significant increase in the fluorescence intensity of both phospho-eEF2 kinase **(D)** and phospho-eEF2 **(E)** in growth cones treated with a DMSO vehicle control (ctrl). However, no significant changes in fluorescence intensity following netrin-1 stimulation were observed following inhibition of the microtubule cytoskeleton with colchicine (colc; **(D, E)**). The number of growth cones analyzed in each treatment group can be found in the corresponding bar of the respective graphs. ns = not significant. ***P < 0.0005 Mann-Whitney test. Scale bar 10 μm.

Translation occurs in three phases: initiation, elongation, and termination. Initiation is the most common step of translation regulation, when the ribosome is recruited to the mRNA, but the polypeptide chain elongation step can also serve as a point of regulation. Since microtubules are not required for translation initiation (no effect on eIF4E-BP1 phosphorylation), we asked whether they regulate the remaining steps, such as phosphorylation of eukaryotic elongation factor 2 (eEF2) by eEF2 kinase (eEF2K) [42] which is downstream of the mTOR protein complex mTORC1 [43]. In whole mammalian cells and tissues, eEF2K phosphorylates and inactivates eEF2 leading to an arrest in mRNA translation [44-46], yet in dendrites, activation of eEF2K is associated with increased synthesis of specific proteins [47-51]. We therefore assessed the activation of eEF2K in growth cones using QIF with an anti-phospho-EF2K antibody (ser366). Growth cones in basal conditions (no netrin-1) exhibited a positive phospho-EF2K signal that was significantly reduced following inhibition of mTOR activity with rapamycin (Figure 5A, B, C), consistent with previous studies showing mTORC1-sensitive eEF2K activation [52]. Stimulation with netrin-1 for 5 min elicited a significant increase in both phospho-eEF2K and phospho-eEF2 signals in growth cones, and these increases were prevented by treatment with colchicine (Figure 5D, E). Cytochalasin D treatment also inhibited activation of both eEF2K and eEF2 (data not shown), although this is not surprising given that F-actin is needed for the upstream steps of the pathway (as shown above). The possibility that the F-actin cytoskeleton is also involved in regulating eEF2K, however, cannot be eliminated. Thus, our results show that the activation of the eEF2K/eEF2 pathway is dependent on microtubules (and possibly F-actin) but leave open the determination of whether increased polypeptide elongation or the prevention of its arrest requires microtubule function.

### Microtubule disruption depletes RNP granules from the growth cone periphery

**Figure 6:**
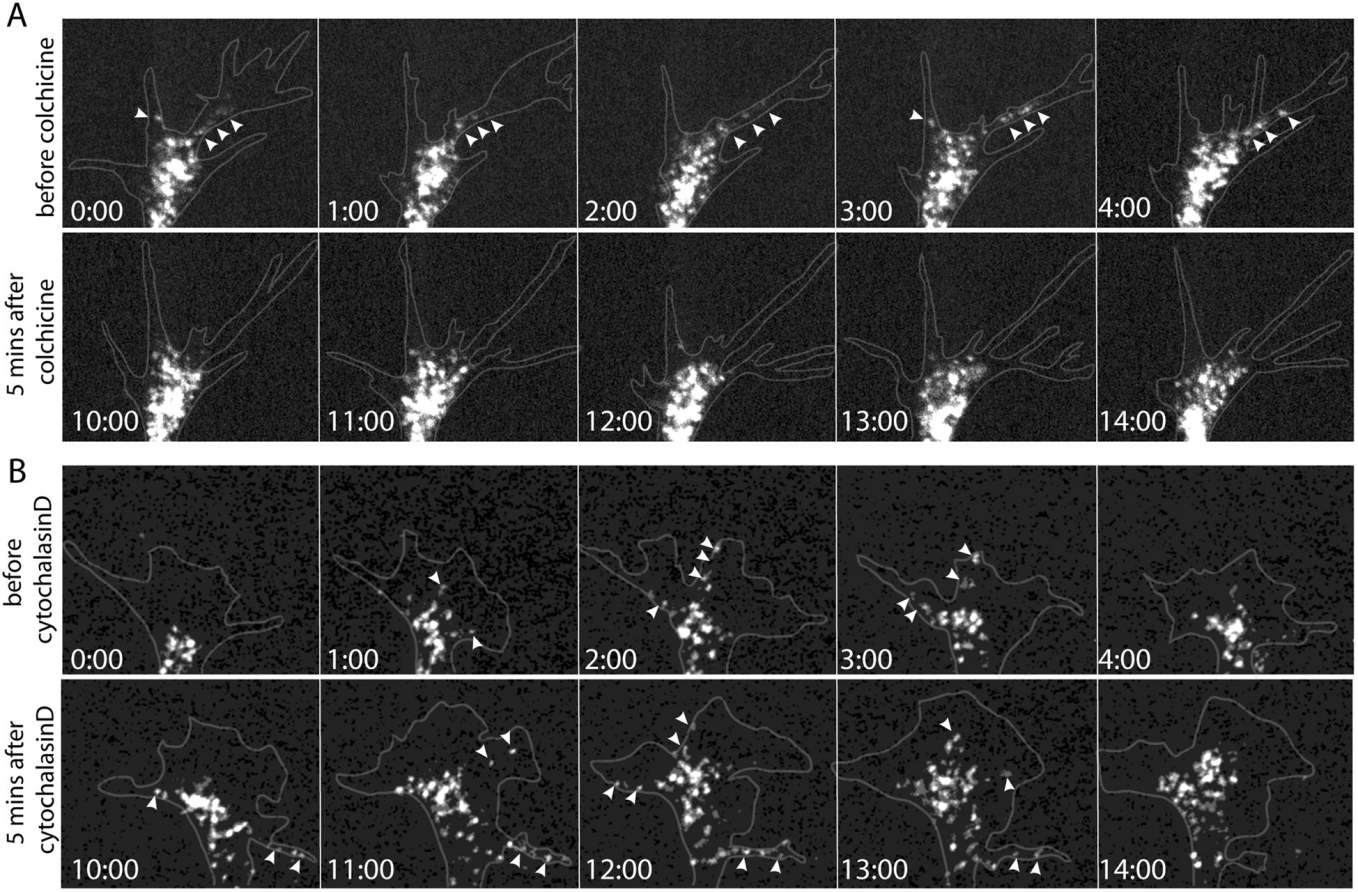
**Inhibition of dynamic microtubules prevents RNP granule movement into the growth cone periphery.** Embryos were injected with fluorescently labeled UTP (Cy3-UTP) at the four to eight-cell stage to visualize ribonucleoprotein (RNP) granules. Cultured stage 24/25 retinal explants positive for Cy3-UTP-labeled RNP granules were selected for time-lapse imaging on an inverted spinning disk confocal system (60× water immersion objective, 5-s acquisition interval) and treated on-stage with cytoskeletal disrupting drugs. Prior to colchicine treatment, RNP granules were trafficked to the growth cone periphery (arrowheads, top row in **(A)**). However, colchicine treatment acutely blocked RNP granule movement into the peripheral domain (bottom row in **(A)**, 8 out of 10 growth cones). In contrast, cytochalasin D treatment did not affect RNP granule trafficking to the periphery (arrowheads in bottom row of **(B)**, 8 out of 11 growth cones). Representative frames per minute from before and 5 min after colchicine (panels in **A**) or cytochalasin D (panels in **B**) treatment are shown. Numbers in the lower left of each image represent time in minutes.

Since mRNAs are actively transported into distal axons on microtubules via motor proteins [17,53,54], we hypothesized that microtubules might also be required for RNP trafficking into the growth cone periphery, in addition to their role in facilitating polypeptide elongation. To investigate this, we labeled ribonucleoprotein (RNP)-containing granules in *Xenopus* retinal ganglion cells by injecting fluorescently conjugated uridine triphosphate (Cy3-UTP) analogs into dorsal blastomeres of four- to eight-cell stage embryos. Eye primordia of stage 24/25 embryos were cultured on coverslips, and growth cones containing Cy3-UTP-labeled granules were imaged before and after addition of cytoskeletal-acting drugs (Figure 6A, B). Time-lapse imaging revealed that the Cy3-UTP-positive RNP granules were highly dynamic in the axon moving anterogradely and retrogradely. In the growth cone, Cy3-UTP-positive RNP granules accumulated primarily in the central domain and individual granules commonly made excursions into lamellipodia and filopodia in the growth cone periphery before cytoskeletal drug treatment (Figure 6A, B, top panels, arrowhead; Movie 1). This dynamic movement of RNP granules into the periphery was greatly reduced in growth cones treated with colchicine (Figure 6A, lower panel, 8 growth cones from a total of 10 growth cones exhibited a reduction in the movement of RNP granules into the periphery; Additional files 1 and 2) yet persisted in cytochalasin D-treated cells (Figure 6B lower panel, arrowhead, 8 growth cones from a total of 11 growth cones exhibited continued movement of RNP granules into the periphery; Additional files 3 and 4). The number of RNP granules from −5 to 0 min (before drug addition) and 5 to 10 min after drug addition in the growth cone periphery was also assessed by counting the number of RNP granules in the growth cone periphery at 1-min intervals. The average number of peripherally located granules per colchicine-treated growth cone was 10.5 ± 2.4 and 1.4 ± 0.3 5 min before and 5 min after drug treatment, respectively. On the other hand, the number of peripherally located RNP granules in cytochalasin D-treated growth cones was 9.5 ± 2.2 and 10.2 ± 2.3, before and after drug treatment, respectively. We conclude that dynamic microtubules, but not actin, are required for delivery of RNP granules to the growth cone periphery. Colchicine, by blocking the netrin-1-induced re-localization of RNPs into the growth cone filopodia/lamellipodia [5], could reduce the availability of a population of mRNAs to activated ribosomal machinery in the periphery and thereby reduce translation.

## Discussion

Guidance cue-induced protein synthesis in the growth cone plays a role in acute steering responses, such as turning, and many of the locally translated proteins are involved in growth cone motility [4,5,7,55]. Genome-wide profiling and candidate studies have shown that the mRNAs of several cytoskeletal components and their regulators are present and locally translated in growth cones in response to guidance cues, but as the cytoskeleton may itself regulate translation [56], we investigated the potential roles of the two major growth cone cytoskeletal systems, F-actin and microtubules, in the control of cue-induced LPS in growth cones. We found that both cytoskeletal systems are essential for cue-induced LPS but that they are involved at different stages in the translation process. An intact actin cytoskeleton is required for netrin-1-induced activation of translation initiation while microtubules are needed for polypeptide chain elongation and the delivery of RNPs to their correct target sites.

In line with previous studies showing a requirement for the actin cytoskeleton in translation [57,58], our findings demonstrate that an intact F-actin network is crucial for LPS in axonal growth cones in response to attractive guidance cues such as netrin-1. Indeed, our results reveal that proper actin dynamics are required at least as early as PI3K/Akt activation after netrin-1 stimulation. The resolution of the spinning disk confocal was not sufficient to allow us to determine whether the PHAkt-GFP biosensor was associated with the cellular membrane. However, the fact that this is the known site of Akt activation suggests that the decline in the biosensor (PHAkt-GFP) signal in the netrin-1 stimulated growth cones in the presence of cytochalasin D was due either to the failure of the reporter or its activators to translocate to the membrane [59]. Our results are also consistent with findings that implicate the F-actin cytoskeleton in the control of several aspects of translational control, such as translation initiation and anchoring of the elongation factor 1α to the F-actin network [57,60]. Moreover, mRNA translation itself has been shown to require the integrity of the F-actin network in models as diverse as yeast [57] and goldfish Mauthner axons [58].

Accumulating evidence implicates the F-actin network as a platform on which translation initiation is regulated [61]. Using both morphological and biochemical techniques, mRNAs, polysomes, and translational machinery have been localized to the cytoskeleton [62]. More recently, the F-actin-binding protein Short stop (Shot) and Krasavietz, a Shot binding partner, have been shown to provide a direct link between cytoskeletal reorganization and translation regulation in midline crossing of commissural neurons in the *Drosophila* nerve cord [63]. These findings suggest that the F-actin cytoskeleton acts as a coordinating center, enabling the rapid transmission of signals from activated guidance receptors to the translational machinery and hence facilitating the rapid local protein synthesis initiated by guidance cues such as netrin-1 [5]. Although it is not possible from these experiments to determine specifically where F-actin is needed, as it is required from the earliest stages downstream of receptor activation, it seems likely that the F-actin network will be required at multiple levels of this hierarchy, due to the localization of many components of the translational machinery to the actin network.

Our findings show that intact microtubules are also essential for axonal LPS. However, although F-actin and microtubules together coordinately regulate axon extension/retraction, our results reveal that the early steps of translation (eIF4E-BP1 activation) occur in the absence of intact microtubule cytoskeleton. What role, then, do microtubules play in this process? Previous work has shown that mRNAs are actively transported into the distal axon on microtubules via motor proteins [17,53,54,64]. Furthermore, in neurons, poly(A) mRNA binds to microtubules and ribosomes and several elongation factors are associated with microtubule cytoskeleton [64-68] indicating that microtubules play a role in tethering the translational machinery and potentially regulating translation. In line with these previous studies, our time-lapse imaging results show that RNA trafficking in the growth cone, particularly into the peripheral regions, depends on microtubules and not F-actin. However, since availability of mRNAs is a prerequisite for translation initiation, how can we explain the fact that colchicine did not block netrin-1-induced eIF4E-BP1 phosphorylation? One possibility is that a small population of mRNAs is actively transported to the growth cone upon netrin-1 stimulation but that other mRNAs are already present in the periphery. As colchicine does not remove existing mRNAs, the netrin-1 stimulus may be sufficient to initiate the translation machinery on these mRNAs, explaining the persisting phospho-eIF4E-BP signal in such treatments.

Our results show that an intact microtubule cytoskeleton is needed for netrin-1’s activation of eEF2K implicating a role in regulating the elongation or arrest steps of translation. Classically, eEF2K phosphorylates and inactivates eEF2 leading to an arrest in mRNA translation [44-46]. However, we found that 5 min of netrin-1 stimulation triggers an increase in both protein synthesis and eEF2K activation in axonal growth cones. This result is more in line with recent work in dendrites where eEF2K is activated by glutamate signaling through both NMDA and glutamate metabotropic receptors [47,48]. In this system, the translation rate of specific mRNAs involved in synapse function such as Arc, MAP1B, and alphaCaMKII, is upregulated [49-51]. This raises the intriguing idea that activation of the eEF2k/eEF2 pathway by netrin-1 may repress global translation while at the same time upregulating translation in specific subcellular compartments such as dendrites and growth cones where select mRNAs such as β-actin, GAP43, and Arc play highly localized roles in growth, guidance, and synaptic function.

In the context of axon guidance, actin pharmacological inhibitors cause axon pathfinding errors *in vivo* [69,70]. Since cytochalasin treatment leads to the loss of the actin-rich filopodia from growth cones, these studies concluded that filopodia are necessary for accurate pathfinding [69,70]. Puzzlingly, however, growth cones can pathfind normally when their filopodia are eliminated by Ena/VASP depletion rather than by cytochalasin [71]. Our current findings offer a possible explanation for these apparently discrepant findings in terms of axonal translation. Ena/VASP depletion should not affect the integrity of the F-actin cytoskeleton, only its polymerization. Thus, in addition to essential roles, it plays in growth cone motility and structure, F-actin also provides an essential platform for LPS allowing the spatial regulation of the local proteome in subcellular space, which may be important for cue-induced turning [5]. Our data on the role of actin in the initiation of translation due to netrin-1 signaling is interesting in light of the results of Tcherkezian *et al*. [29], who found that DCC, the netrin receptor, controls translation of axonally localized mRNAs by directly interacting with translation initiation factors as well as ribosomal proteins. If, as this model suggests, DCC acts as a platform for the assembly of translation regulators, disruption of the cytoskeleton may interfere with the translocation of these factors to this platform.

## Conclusions

In summary, we have shown that both an intact actin and microtubule cytoskeleton are essential for cue-induced LPS, but that the two elements have distinct roles in this process. Only disruption of actin blocks the early steps in translation initiation. In both cases, there is still much to learn about the molecular nature of these dynamic interactions, which particular steps are affected, and how exactly these are regulated by the different cytoskeletal components. Further dissection of the relation between cytoskeletal elements and translation machinery may help explain the logistics of translation control in axon navigation.

## Methods

### Primary culture of *Xenopus* retinal explants

Oocytes obtained from adult wild-type female *Xenopus laevis* injected with human chorionic gonadotropin hormone (Sigma-Aldrich Corporation, St. Louis, MO, USA) were fertilized *in vitro,* then raised at 14°C to 18°C in 0.1× modified Barth’s saline (MBS). Embryos were staged according to the tables of Nieuwkoop and Faber. Eye primordia from stage 24/25 embryos were dissected and cultured on acid-cleaned 12-mm round glass coverslips coated with 10 μg/ml poly-L-lysine (Sigma-Aldrich Corporation, St. Louis, MO, USA) and 1 μg/ml fibronectin (Calbiochem-Novabiochem Corp., La Jolla, CA, USA). Cultures were kept in 60% L15 medium (Invitrogen Life Technologies, Carlsbad, CA, USA) supplemented with Pen/Strep/Fungizone (Invitrogen Life Technologies, Carlsbad, CA, USA, 1:100) for 24 h at 20°C before experimentation. All animal experiments were approved by the Ethical Review Committee of the University of Cambridge and complied with Home Office guidelines.

### Pharmacological agents

The following pharmacological agents were bath applied to retinal cultures for 5 min to interfere with actin and microtubule dynamics: 0.1 μM cytochalasin D (Sigma-Aldrich Corporation, St. Louis, MO, USA), 30 nM latrunculin B (Sigma-Aldrich Corporation, St. Louis, MO, USA), 12.5 μM colchicine (Sigma-Aldrich Corporation, St. Louis, MO, USA), and 0.1 μM nocodazole (Sigma-Aldrich Corporation, St. Louis, MO, USA). Cycloheximide (100 μg/ml) (Sigma-Aldrich Corporation, St. Louis, MO, USA) and 40 μM anisomycin (Sigma-Aldrich Corporation, St. Louis, MO, USA) were used to block protein synthesis. DMSO (0.1%) (vehicle) was included in control conditions.

### Antibodies

Phosphorylated and total forms of proteins were detected by immunocytochemistry using antibodies from the following companies: anti-phospho-eIF4E-BP1 (Ser65) (1:100), anti-eIF4E-BP1 (1:100), anti-mTOR (1:100), anti-phospho-Akt (Ser473) (1:250), anti-Akt (1:250), anti-phospho-eEF2 (Thr 56) (1:100), anti-eEF2 kinase (1:100), and anti-phospho-eEF2 kinase (Ser366) from Cell Signaling Technology, Danvers, MA, USA; anti-phospho-mTOR (S2448) (1:250) from Abcam, Cambridge, MA, USA; and anti-EF2 (C-14) (1:100) from Santa Cruz Biotechnology, Santa Cruz, CA, USA. F-actin was visualized with Alexa-Fluor 594-phalloidin (used at 1:50; Invitrogen Life Technologies, Carlsbad, CA, USA), and β-tubulin was labeled with anti-β-tubulin (used at 1:1000; Sigma-Aldrich Corporation, St. Louis, MO, USA). Secondary antibodies conjugated with Alexa Fluor dyes were from Invitrogen Life Technologies, Carlsbad, CA, USA.

### Immunofluorescence and image acquisition

Twenty-four-hour cultures of stage 24/25 retinal explants were incubated with cytoskeletal inhibitors for 5 min, followed by 5 min of netrin-1 (600 ng/ml, mouse recombinant, R&D Systems, Minneapolis, MN, USA) or control medium containing netrin-1 carrier 0.1% bovine serum albumin (BSA; Sigma-Aldrich Corporation, St. Louis, MO, USA). Cultures were fixed in 2% paraformaldehyde/7.5% sucrose for 30 min, permeabilized with 0.1% saponin (Sigma-Aldrich Corporation, St. Louis, MO, USA), and blocked with 5% goat serum. The cultures were then labeled with primary antibodies for 1 h, followed by 30 min of secondary antibody incubation (Alexa Fluor-conjugated, Invitrogen Life Technologies, Carlsbad, CA, USA, 1:500), and mounted in FluorSave (Calbiochem-Novabiochem Corp., La Jolla, CA, USA). Non-collapsed growth cones were visualized with a 60× 1.4 NA oil objective on a Nikon Eclipse inverted microscope (Nikon, Tokyo, Japan). Using phase optics to avoid biased selection of fluorescence, individual growth cones were randomly selected and imaged using a Hamamatsu digital CCD camera (Hamamatsu Photonics, Hamamatsu, Japan). A fluorescent image was then captured, exposure time being kept constant and below greyscale pixel saturation.

### Quantification of fluorescence intensity

For quantification of fluorescence intensity, the growth cone outline was traced on the phase image by using Openlab software (Improvision, Lexington, MA, USA) and then superimposed on the fluorescent image. The software calculated the fluorescent intensity within the growth cone, giving a measurement of pixel intensity per unit area. The growth cone outline was then placed in an adjacent area clear of cellular material to record the background fluorescent intensity. This reading was subtracted from the growth cone reading, yielding the background-corrected intensity. The fluorescent intensities of between 25 and 60 growth cones per sample group in each experiment were collected. Data were normalized against the control-treated group to allow for comparisons between experiments and are presented as percentage of control fluorescent intensity. Error bars represent SEM. Samples from independent experiments were stained, imaged, and processed under identical conditions. Counts in which the experimenter was blind to the identity of the samples resulted in similar results. Statistical analysis was performed using a two-tailed Mann-Whitney test.

### Protein synthesis metabolic assays

To measure protein synthesis in isolated growth cones, the metabolic assays ^3^H-leucine incorporation and AHA protein synthesis fluorescence microscopy were performed. ^3^H-leucine incorporation assays were performed as described previously [53]. Briefly, stage 35/36 retinal explants containing retinal ganglion cell bodies were removed from the neurites with a razor. Coverslips with attached neurites were moved to leucine-free medium, and pharmacological inhibitors were added 5 min before the addition of control medium or netrin-1, immediately followed by the addition of L-[4,5-^3^H]leucine (5.66 TBq/mmol; Amersham). Cultures were then rinsed and fixed in 25% tri-chloroacetic acid, and 3H uptake was measured with a scintillation counter. We performed this experiment on four independent occasions, and within each trial, experiments were performed in duplicate. The AHA assay was performed following instructions from the Click-iT AHA Alexa Fluor 488 Protein Synthesis HCS Assay kit (Invitrogen Life Technologies, Carlsbad, CA, USA, C10289), which detects incorporated methionine analog AHA with an Alexa Fluor 488 alkyne using Click chemistry [18]. Culture medium was replaced with methionine-free medium followed by 20 min of 100-μM AHA reagent incubation. Cytoskeletal inhibitor was applied for 5 min, followed by 5 min of netrin-1 (600 ng/ml) or control (0.1% BSA). The cells were then fixed with 2% paraformaldehyde/7.5% sucrose and permeabilized with 0.1% Triton-X-100. Click-iT reaction mix was added to the cultures for 30 min, and samples were mounted using FluorSave mounting medium (Calbiochem-Novabiochem Corp., La Jolla, CA, USA) after a final wash.

### Live cell imaging in *Xenopus* retinal ganglion neuron growth cones

For live cell imaging of PI3K activity, *Xenopus* embryos were injected at the four- to eight-cell stage in dorsal blastomeres with 125 pg of DNA encoding PHAkt-GFP biosensor (Addgene plasmid 18836, kindly deposited by Dr. Craig Montell [72]) per blastomere. For labeling RNP granules, four- to eight-cell stage embryos were injected with 10 nl of 100 μM Cyanine 3-UTP (Perkin Elmer, Waltham, MA, USA). In order to co-image RNP granules with microtubules, some embryos were co-injected with 100 pg per blastomere of DNA encoding tubulin-EGFP (pEGFP vector, Clonetech, Palo Alto, CA, USA). Stage 24/25 embryos with positive fluorescence labeling in the eye primordia were selected for culture on glass bottom imaging dishes (MatTek). Culture medium was replaced with imaging medium (60% Phenol Red-free L15 medium supplemented with 1 mg/ml BSA, 1 mg/ml L-carnosine, and 25 mM vitamin E; chemicals from Sigma-Aldrich Corporation, St. Louis, MO, USA or Calbiochem-Novabiochem Corp., La Jolla, CA, USA) 1 h before microscopy. Label-positive growth cones were imaged on an inverted spinning disk confocal system (Perkin Elmer UltraVIEW ERS, Waltham, MA, USA) using a 60× 1.2 NA water immersion objective at room temperature (23°C). Time-lapse images were taken for 5 min before and after cytoskeletal drug application and continued for 10 to 15 min after netrin-1 bath stimulus at 5-s acquisition intervals using Volocity software (Perkin Elmer, Waltham, MA, USA). Fluorescence intensity analysis was done in ImageJ software (NIH), and intensity map figures were generated with MATLAB 7.14 (release R2012a, Mathworks).

## Additional files

Additional file 1:
**Inhibition of dynamic microtubules prevents RNP granule movement into the growth cone periphery.** Movie revealing that inhibition of dynamic microtubules inhibits the movement of RNP granules to the growth cone periphery. Embryos were injected with fluorescently labeled UTP (Cy3-UTP) at the four to eight-cell stage to visualize ribonucleoprotein (RNP) granules. Cultured stage 24/25 retinal explants positive for Cy3-UTP-labeled RNP granules were selected for time-lapse imaging on an inverted spinning disk confocal system (60× water immersion objective, 5-s acquisition interval) and treated on-stage with the microtubule disrupting drug colchicine (colch). Prior to colchicine treatment, RNP granules were trafficked to the growth cone periphery. However, colchicine treatment acutely blocked RNP granule movement into the peripheral growth cone domain. The corresponding differential interference contrast (DIC) images for this time-lapse sequence is provided in Additional file 2. Additional file 2:
**Inhibition of dynamic microtubules prevents RNP granule movement into the growth cone periphery.** Differential interference contrast imaging of the growth cone shown in Additional file 1, revealing that this growth cone does possess cytoplasmic extensions following treatment with colchicine, suggesting that the loss of RNP granule movement to the growth cone periphery following microtubule disruption is not due to the loss of these processes. Additional file 2 is taken with differential interference contrast (DIC) optics (5-s acquisition interval). Additional file 3:
**Inhibition of the actin cytoskeleton does not prevent RNP granule movement into the growth cone periphery.** Movie revealing that inhibition the actin cytoskeleton does not inhibit the movement of RNP granules to the growth cone periphery. Embryos were injected with fluorescently labeled UTP (Cy3-UTP) at the four to eight-cell stage to visualize ribonucleoprotein (RNP) granules. Cultured stage 24/25 retinal explants positive for Cy3-UTP-labeled RNP granules were selected for time-lapse imaging on an inverted spinning disk confocal system (60× water immersion objective, 5-s acquisition interval) and treated on-stage with the actin disrupting drug cytochalasin D. Prior to colchicine treatment, RNP granules were trafficked to the growth cone periphery, a process that continued following cytochalasin D treatment. The corresponding differential interference contrast images for this time-lapse sequence is provided in Additional file 4. Additional file 4:
**Inhibition of the actin cytoskeleton does not prevent RNP granule movement into the growth cone periphery.** Differential interference contrast imaging of the growth cone shown in Additional file 3, revealing that this growth cone possesses cytoplasmic extensions following treatment with cytochalasin D. Additional file 4 is taken with differential interference contrast (DIC) optics (5-s acquisition interval).

## Abbreviations

3′ UTR: 3′ untranslated region
AHA: L-azidohomoalanine
eEF2: eukaryotic elongation factor 2
eEF2K: eEF2 kinase
eIF4E-BP1: translation initiation factor regulator eIF4E-binding protein 1
FUNCAT: fluorescent non-canonical amino acid tagging
LPS: local protein synthesis
mTOR: target of rapamycin
PI3K: phosphoinositide 3-kinase
QIF: quantitative immunofluorescence
RGC: retinal ganglion cell
RNP: ribonucleoparticle
ZBP: zipcode binding protein 1
