# Erratum to: Differential requirement of F-actin and microtubule cytoskeleton in cue-induced local protein synthesis in axonal growth cones

**DOI:** 10.1186/s13064-015-0043-9

**Published:** 2015-06-19

**Authors:** Michael Piper, Aih Cheun Lee, Francisca P.G van Horck, Heather McNeilly, Trina Bo Lu, William A Harris, Christine E Holt

**Affiliations:** Department of Physiology, Development and Neuroscience, University of Cambridge, Downing street, Cambridge, CB2 3DY UK; Current address: The School of Biomedical Sciences and the Queensland Brain Institute, The University of Queensland, St Lucia, 4072 QLD Australia; Current address: Institute of Neuroscience, Chinese Academy of Sciences, Shanghai, 200031 China

## Erratum

We were recently made aware that one of the figures (Figure four (Fig. 1 here)) in our manuscript detailing the contribution of the cytoskeleton to cue-induced protein synthesis [1] contains a minor formatting error, introduced while the panels of the figures were being compiled. In panel I of the original Figure four (Fig. 1 here), we used white lines to divide the images of the growth cones that had been exposed to different cytoskeletal disrupting agents. However, the white lines were not placed appropriately, and as such, some of the filopodia from the control growth cones (the two left hand growth cones in panel I of Figure four (Fig. 1 here)) can be seen to the right of the dividing line. We have amended this figure to avoid any potential misinterpretation of the data.

**Fig. 1:**
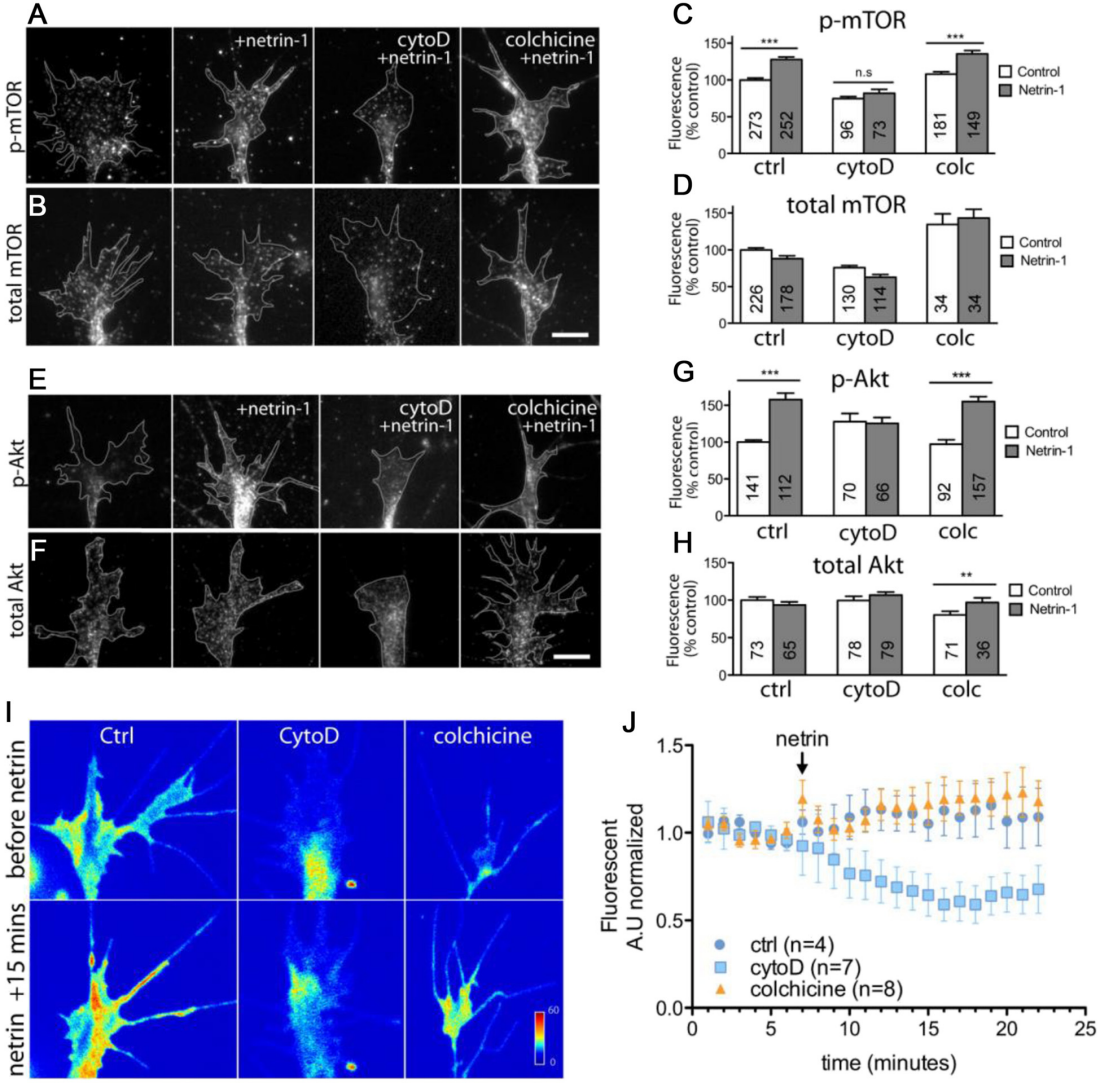
Actin, but not microtubule, disruption blocks netrin-1 induced PI3K/Akt/mTOR signaling in growth cones. Cultured stage 24/25 retinal explants were treated with a DMSO vehicle control, cytochalasin D (CytD), or colchicine (Colc) for 5 min, followed by 5-min stimulation with either control medium or netrin-1. Quantitative immunofluorescence showed that levels of activated mTOR (p-mTOR; (**a**)) and Akt (p-Akt; (**e**)) were elevated by netrin-1 stimulation, a process that was prevented by actin, but not microtubule disruption (quantified in (**c**) and (**g**)). Total levels of mTOR (**b**) and Akt (**f**) were not significantly altered following stimulation with netrin-1, in either untreated or growth cones treated with cytoskeletal disrupting agents (quantified in (**d**) and (**h**)), with the exception of total Akt levels in growth cones treated with colchicine, which showed a small, but significant increase in fluorescence intensity. The number of growth cones analyzed in each treatment group can be found in the corresponding bar of the respective graphs. **P < 0.005; ***P < 0.0001 Mann–Whitney test. (**i**) Pseudocolored images of a live PI3K biosensor (PHAkt-GFP) before and after netrin-1 stimulation in the presence of cytoskeletal inhibitors. Growth cones expressing low levels of PHAkt-GFP were treated with cytoskeletal inhibitors (0.1 μM cytochalasin D or 12.5 μM colchicine) on stage during time-lapse acquisition on an inverted spinning disk confocal system (60× water immersion). Time-lapse imaging showed an increase in PHAkt-GFP signal after netrin-1 stimulation, which was inhibited by cytochalasin D treatment, but not affected by colchicine treatment (**j**). Background-subtracted fluorescent signals were normalized to the control medium at time 0. Scale bar 10 μm
